# Advances in EV isolation technology and function 

**DOI:** 10.20517/evcna.2021.09

**Published:** 2021-03-30

**Authors:** Y. Peng Loh

**Affiliations:** American Biochemist and Molecular Biologist, Bethesda, MD 20817, USA.

I would like to introduce to you our second issue of *EVCNA*. In this issue we have assembled 5 articles which represent the breadth of our journal. They include 2 reviews and 1 research article covering new technologies in extracellular vesicle (EV) isolation, production and characterization. Dr. Choi’s^[1] ^group reviews new platforms in the production of therapeutic exosomes from human cell lines. A review from Dr. Soper’s^[2] ^team discusses the pros and cons of traditional and non-traditional, including microfluidics and resistive pulse sensing technologies for extracellular vesicle isolation and detection. A paper from Dr. Baccarelli’s^[3] ^lab presents new procedures for the isolation and characterization of extracellular vesicles from saliva of children with asthma, which will facilitate the use of saliva exosomes as biomarkers in the future. Additionally, in this issue, Dr. Holliday’s^[4] ^lab has contributed an interesting review focusing on direct communication between extracellular vesicles in osteoblasts/osteocytes with osteoclasts in bone remodeling, offering a thought-provoking prospect in the field. Finally, a review from Dr. Loh’s^[5]^ group discusses the function of exosomes in various neurological disorders and brain cancer, highlighting many potential biomarkers, especially miRNAs, in serum - derived exosomes associated with these diseases that can be used as non-invasive diagnostic tools, since exosomes cross the blood brain and conversional biopsy is not possible. This issue also contains a report by Dr. Ikezu^[6] ^on exciting papers presented at the Alzheimer’s/Parkinson’s Disease (ADPD) 2021 conference, which included the use of blood and CSF-derived exosomes in liquid biopsy to diagnose brain atrophy and cognitive dysfunction, and identification of specific EV cargoes from patients associated with various neurological deficits. Readers will find the articles in this issue especially useful in updating the current knowledge of EV research in many directions from understanding the pathophysiological roles of exosomes to new isolation technologies and potential applications of EVs in therapies.

## DECLARATIONS

### Copyright

©The Author(s) 2021.

## References

[1] Kim J, Song Y, Park CH, Choi C (2021). Platform technologies and human cell lines for the production of therapeutic exosomes. Extracell Vesicles Circ Nucleic Acids.

[2] Zhao Z, Wijerathne H, Godwin AK, Soper SA (2021). Isolation and analysis methods of extracellular vesicles (EVs). Extracell Vesicles Circ Nucleic Acids.

[3] Comfort N, Bloomquist TR, Shephard AP, Petty CR, Cunningham A, Hauptman M, Phipatanakul W, Baccarelli A (2021). Isolation and characterization of extracellular vesicles in saliva of children with asthma. Extracell Vesicles Circ Nucleic Acids.

[4] Holliday LS, Patel SS, Rody WJ Jr (2021). RANKL and RANK in extracellular vesicles: surprising new players in bone remodeling. Extracell Vesicles Circ Nucleic Acids.

[5] Xiao L, Hareendran S, Loh YP (2021). Function of exosomes in neurological disorders and brain tumors. Extracell Vesicles Circ Nucleic Acids.

[6] Ikezu T (2021). Current Trends in Extracellular Vesicle Research on Neuroscience from ADPD2021 Meeting. Extracell Vesicles Circ Nucleic Acids.

